# Molecular investigation of possible relationships concerning bovine leukemia virus and breast cancer

**DOI:** 10.1038/s41598-022-08181-5

**Published:** 2022-03-09

**Authors:** Zanib Khan, Muhammad Abubakar, Muhammad Javed Arshed, Roohi Aslam, Sadia Sattar, Naseer Ali Shah, Sundus Javed, Aamira Tariq, Nazish Bostan, Shumaila Manzoor

**Affiliations:** 1 Biosciences Department, COMSATS University, Islamabad, Pakistan; 2 National Veterinary Laboratory, Park Road, Islamabad, Pakistan; 3 NUTECH School of Applied Sciences and Humanities, National University of Technology, Islamabad, Pakistan

**Keywords:** Cancer, Microbiology

## Abstract

Worldwide, breast cancer has an eminent morbidity and mortality rate, as it is a neoplastic disease among females. The query of the prospective danger of bovine leukemia virus (BLV) to humans is an old but exceedingly topical focus of scientific debate. The objective of the current study was to determine the possible relationship between BLV and breast cancer. A total of 2710 formalin-fixed paraffin-embedded (FFPE) breast cancer samples were selected regardless of the age, ethnicity, or municipality origin of the subjects. The presence of BLV in human breast cancer was determined through nested PCR by amplifying *tax* and *gag* genes followed by partial sequencing. Homology was confirmed by using the online BLAST Tool. BLV genes were found to be positive in 26.8% (728/2710) of the samples from breast cancer patients and 10% (10/80) of the samples without cancer (negative control). The results indicated a correlation between the presence of the BLV gene and breast cancer (*odds ratio* = 0.3889; confidence interval = 1,18; p = 0.0029). The current findings suggest a possible link between BLV and human breast carcinoma. Therefore, screening cattle herds and milk products is suggested to reduce the viral transmission risk to humans.

## Introduction

Can a virus be able to cause breast carcinoma? Various researchers have attempted to address this issue for the last 40 years, even a long time since the development of mammary malignant growth in mice through milk-borne infection. Certainly, breast cancer is a complex sickness with various threat factors adding to the ultimate onset of the disease^1,2^. Bovine Leukimia Virus has probable risk to animals and human health. The contradiction about the zoonotic potential of the BLV had not been yet cleared^3^ and there is need to explore its relationship with humans and in order to decipher this anonymity there are several studies to establish relationship of breast cancer and the BLV^4^. Moreover genome fragment of the BLV from the breast cancer patients had been identified but the exact etiology has not been properly declared up till now^5^. There are some of the studies showing the capability of the BLV to infect the mammary cells of the human in-vitro^6,7^ and the antibodies of the BLV had been detected from the human blood^4,8,9^ suggesting that the threat related to BLV accusation and propagation in human is not a matter of negligence^4,10,11^. In 2014, BLV DNA was detected from the breast tissue of humans^11^ and a case–control study in an association of BLV with healthy and cancerous breast tissue was reported^10^. Moreover, one of the recent studies in Panama showed 59% of the cancerous samples and 38% of the precancerous samples by identifying the *gag* gene^12^. Thus, these initial findings are important to explore the zoonosis of BLV. However, there are some controversial results from different countries, as the linkage of breast cancer and BLV was not found in malignant and benign cancer samples *insilico*^13,14^ and in China, the researcher could not find the BLV gene segment from the breast cancer samples, nor was any positive serological test found^15^. Furthermore, the study of Gillet et al. also failed to recover the gene segments of the virus through whole-genome sequencing^16^.

There are various types of breast cancer and which cells in the breast become cancerous determines the type of breast cancer. Breast cancer can begin in several locations in the breast^17^. Breast cancer is usually categorized into two types by WHO *i.e*., noninvasive (in situ) and invasive cancer. Growth within the ducts without penetration of the basement membrane, and hence without the ducts engaging the stroma, is known as in situ carcinoma^18,19^. The most frequent type of non-invasive breast cancer (90%) is ductal carcinoma in situ (DCIS), which is limited to the ducts of the breast. Lobular carcinoma in situ (LCIS) is a less common type of breast cancer that is thought to be a risk factor for the disease. LCIS (lobular neoplasia, lobular carcinoma in situ) is a rapid rise in the number of cells in the milk glands (lobules) of the breast. Breast cancers caused by ILC account for 10% to 15% of all cases^20^. Infiltrating ductal carcinoma (IDC) is a kind of breast cancer that began in a milk duct and has migrated to fibrous or fatty tissue outside of the duct. The most common kind of breast cancer, IDC, accounts for more than 80% of all diagnoses. ILC, also known as infiltrating lobular carcinoma, is the second most frequent type of breast cancer diagnosed in the United States, accounting for 10–15% of all detected invasive breast cancers. Invasive mammary carcinoma is a tumor that has both ductal and lobular carcinoma characteristics. It's not two different malignancies, but rather one with characteristics of both major forms of breast cancer^21,22^. Fibroadenomas are benign breast tumor that is composed of glandular and stromal (connective) tissue. Fibroadenomas are most frequent in women in their twenties and thirties, but they can occur at any age after a woman has gone through menopause, they tend to shrink^1^. The tissue in fibrocystic breasts is lumpy or ropelike in texture and referred to as nodular or glandular breast tissues^23^. Granulomatous mastitis (GM) is a benign chronic inflammatory breast illness that affects only a small percentage of women and is a diverse disorder with a wide range of clinical manifestations^24^.

Unfortunately, due to the high cost of processed milk, the majority of the Pakistani population utilizes raw milk in comparison to treated milk. According to the literature, nations with maximum milk utilization have the maximum breast cancer rate^25,26^. The most important purpose of the present study is to determine the existence of BLV in human breast tissues, which is an exogenous B-lymphotropic Delta retrovirus, an oncogenic member of the *Retroviridae* family and is responsible for enzootic bovine leukosis in cattle^27–40^.

## Materials and methods

### Archived and fresh sample selection

The preserved breast cancer samples from 2015 to 2018 were selected from the biobank of Pakistan Institute of Medical Sciences (PIMS) Islamabad and Ayub Medical Complex Abbottabad, Pakistan along with the pathological details. The samples of 2019 were freshly prepared. A total of 3000 samples were collected initially, out of which 2710 samples were selected for further processing. The 290 samples were excluded based on patient consent and improper/missing pathological data. The 80 biopsy samples which have been declared as non cancerous were taken as a negative control from the patients having benign (noncancerous) lumps in their breast.

### Sample collection

A total of 2710 formalin-fixed paraffin-embedded (FFPE) breast samples were collected under the ethical approval of PIMS Islamabad and Ayub Medical Complex Abbottabad, Pakistan, and consent was obtained from patients before surgery for breast cancer. Written informed consent was taken from the guardians of the patients before including the samples in the present study. The FFPE breast cancer samples were selected regardless of the age, ethnicity, or municipality origin of the subjects. The human subject protocol was approved by the Ethics Review Board of (CUI) COMSATS University, Islamabad vide letter # CUI/Bio/ERB/2021/41. All methods were carried out by following relevant guidelines and regulations approve by COMSATS University Islamabad. The details of the sample types are presented in Table 1.

**Table 1:** Types of breast cancer considered for the current study.

S.no	Types of breast cancer	No of samples
1	Fibroadenoma	1527
2	Fibrocystic disease	114
3	Granulamatous mastitis	83
4	Invasive mammary carcinoma	45
5	Invasive ductal carcinoma	872
6	Invasive lobular carcinoma	22
7	Phyllodes tumor	17
8	Total	2710
9	Negative breast cancer	80

### DNA extraction

The extraction of DNA from the FFPE breast tissue specimens was performed by following the phenol/chloroform method as described by Pikor^41^ with a few modifications. DNA yield and quality were analyzed by using a NanoDrop 2000/2000c (Thermo Scientific) spectrophotometer absorbance at 260/280 ratio and the ratio of 1.8 was used as an indicator of purity of all extracted DNA samples.

### Qualitative analysis of DNA

**Figure 1:**
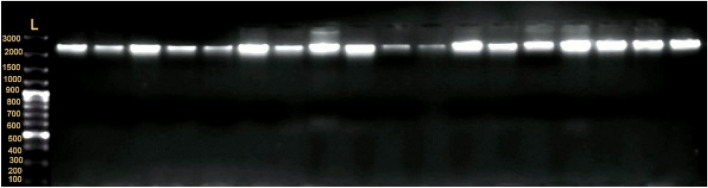
The figure is showing extracted DNA. The lane (L) is the 100 bp DNA ladder and the lanes from 2 to 19 are the bands of the extracted whole-genome DNA of size greater than 2000 bp.

The extracted DNA was run on a 1.5% agarose gel (Merck, CAT#NO:101236), and DNA bands were visualized by using ALPHA-INNOTECT-GEL DOCUMENTATION SYSTEM, as shown in Fig. 1. The house keeping gene Beta-Actin was amplified firstly to ensure the purity of extracted DNA by using Forward 5′-TGGCATCCACGAAACTACCT-3′ and Reverse 5′-TCTCCTTCTGCATCCTGTCG-3′ primers. Briefly 20 ng of template DNA, 50 pmol of forward and reverse primers, 2 × master mix and 10 μl of RNase free water in a final volume of 30 μl reaction mixture was run by initial denaturation at 95 °C for ten minutes, followed by 35 cycles as 95 °C, 59 °C, and 72 °C for 45 s with a final extension at 72 °C for 5 min. Then the PCR product of 134 bp was analysed by using 2% agarose gel. After amplification by the house keeping gene DNA was then amplified by nested PCR for BLV detection.

### Amplification of DNA

A gag and a *tax* gene were amplified through nested PCR (BIO-RAD DNA ENGINE) by using the following set of primers previously used by Buehring^11^ :*Gag* Outer F: 5ʹ-AACACTACGACTTGCAATCC-3ʹ R: 5ʹ-GGTTCCTTAGGACTCCGTCG-3ʹ *Gag* Inner F: 5ʹ-ACCCTACTCCGGCTGACCTA-3ʹ R: 5ʹ-CTTGGACGATGGTGGACCAA-3ʹ and Tax Outer F: 5ʹ-TATTCCACCTCGGCAC-3ʹ R: 5ʹ-ATTGGCATTGGTAGGGCT-3ʹ *Tax* Inner F: 5ʹ-CTTCGGGATCCATTACCTGA-3ʹ R: 5ʹ-GCTCGAAGGGGGAAAGTGAA-3ʹ. The PCR composition and thermocycler optimized conditions are presented in Supplementary Table 1. Following PCR, the fragments were analyzed by using 2% agarose gel electrophoresis. DNA from enzootic bovine leukosis-positive cattle from the Livestock & Dairy Development Department of Peshawar Khyber PakhtunKhwawas used as a positive control.

### DNA sequencing

The resulting PCR products of only two samples amplified by the *gag* gene were sent to the MACROGEN laboratory (Korea) for sequencing. The sequences were compared with those in the GenBank databases (www.ncbi.nlm.nih.gov/blast) by using the BLAST program.

### Statistical analysis

The statistical analyses were performed by using GraphPad Prism software^7^. The relationship between the presence of the BLV gene and breast cancer was analyzed by Fisher's exact test and chi-square tests using GraphPad Prism software.

## Results

### Association of breast cancer with BLV

**Figure 2:**
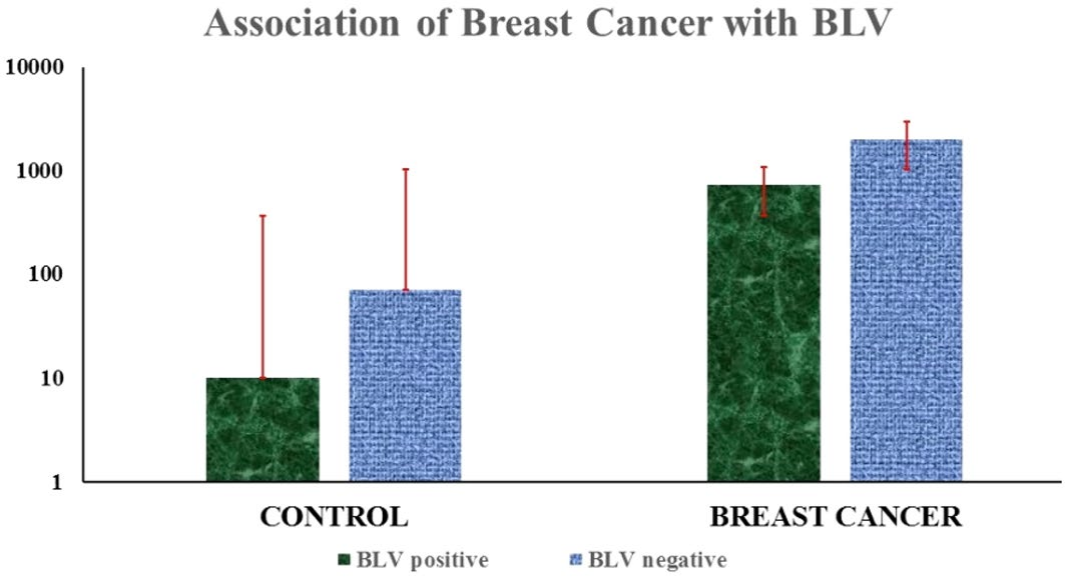
This figure shows the occurrence of BLV in breast cancer. out of the 2710,728 breast cancer samples were found to be positive for BLV, and 1982 were BLV-negative samples.

**Figure 3:**
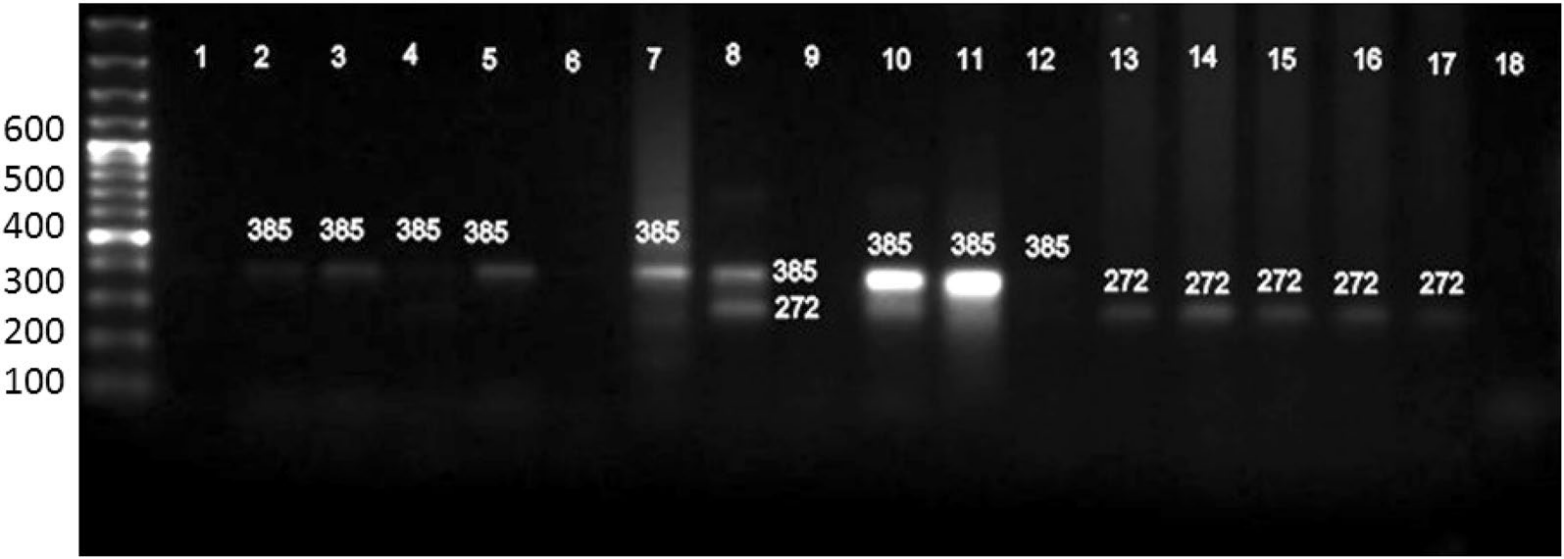
The figure shows the amplified gene fragments of the *gag* outer (385 bp) and *gags* inner genes (272) by nested PCR. Lanes 10, and 11 were the positive controls, while lane 18 was the negative control. Samples 1, 6 and 9 were considered negative, while samples 2, 3, 4, 5, and 7 were positive for both outer and inner *gag* gene amplification.

The association between BLV and breast cancer was analyzed by Fisher's exact test and chi-square tests. BLV genes were found to be positive in 26.8% (728/2710) of the samples from breast cancer patients and 10% (10/80) of the samples without cancer. The current findings indicated a correlation between the presence of the BLV gene and breast cancer (*odds ratio* = 0.3889; confidence interval = 1,18; p = 0.0029). BLV-positive animal DNA was used as a positive control. Out of 2710 FFPE breast cancer tissue samples, 728 (26.8%) were BLV positive by the amplification of 272 bp and 113 bp fragments of the *gag* and *tax* genes (Figs. 2, 3). Out of eighty (n = 80) negative breast cancer samples, 10 (12.5%) were BLV positive. The incidence of BLV post- chemo-subject was 3.5%, while the incidence was found to be almost equal in left- and right-side breast cancer cases, *i.e.,* 50% each.

### Association of BLV with different types of breast cancer

**Figure 4:**
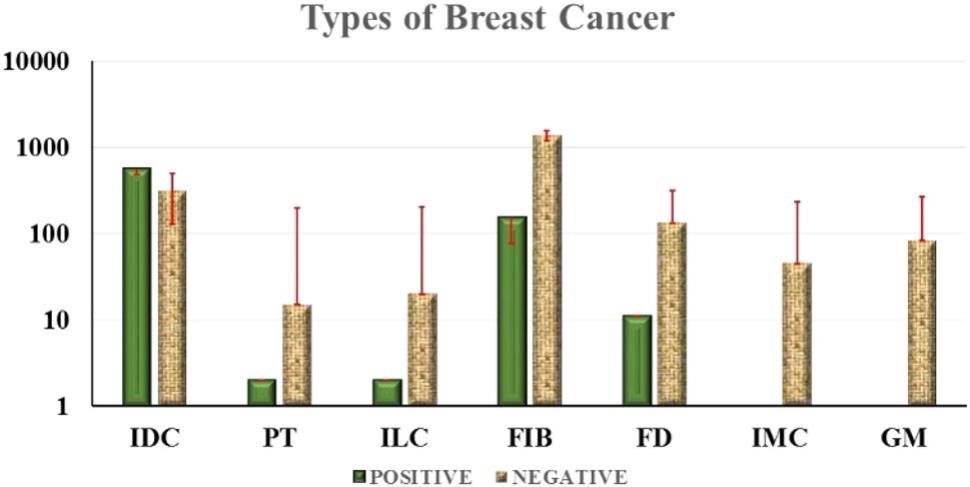
The figure shows the percentage of the prevalence of BLV in different types of breast cancer.

Invasive ductal carcinoma showed the highest prevalence (64%) of the BLV gene, 11.7% Phyllodes Tumor, 10% Fibroadenoma, 9.6% Fibrocystic Disease, and 9% Invasive Lobular Carcinoma. Cancer samples were BLV positive, as shown in the Supplementary Table 2. Ten percent (155) of the fibroadenoma, 9.6% (11) fibrocystic disease, 64% (559) invasive ductal carcinoma, 9% (2) invasive lobular carcinoma, and 11.7% (2) phyllodes tumor cancer samples were BLV positive. All the samples from granulomatous mastitis and invasive mammary carcinoma were negative for the BLV gag and tax genes (Fig. 4).

### Grade of cancer

**Figure 5:**
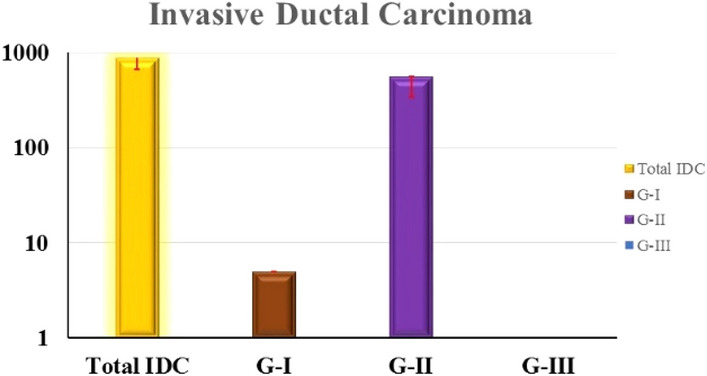
The figure shows the high prevalence of the BLV gene in grade II invasive ductal carcinoma (57%). In contrast, 59/872 (6.7%) of the BLV-positive IDC samples were IDC grade I.

In the current study, invasive ductal carcinoma (IDC) showed the highest prevalence (64%) of the BLV genes. The IDC has generally three grades I, II, and III. The prevalence of BLV genes was mostly found in breast cancer with IDC grade II. Out of 872 positive IDC samples, 500 (57%) belonged to grade II (Fig. 5).

### Statistical analysis

The relationship between the presence of the BLV gene and breast cancer was analyzed by chi-square tests, as shown in Supplementary Table 3. The chi-square statistic was 8.241, and the *p*-value was < 0.00001. The result is significant at *p* < 0.05.

### Sequencing identity comparison by BLAST

The BLV *gag* gene nucleotide sequences amplified in the current study showed 99.56% similarity to the sequences found in GenBank as follows:Bovine leukemia virus isolate G235 gag protein gene, partial cds Sequence ID: MG800837.1, Bovine leukemia virus isolate G225 gag protein gene, partial cds Sequence ID: MG800836.1, MG800834.1, strain: pvAJ029 Sequence ID: AP019593.1, pvAJ028 Sequence ID: AP019592.1, strain: pvAJ027 Sequence ID: AP019591.1, strain: pvAJ026 Sequence ID: AP019590.1, strain: pvAJ025 Sequence ID: AP019589.1, strain pvAJ024 Sequence ID: AP019588.1, strain: pvAJ023 Sequence ID: AP019587.1, strain: pvAJ021 Sequence ID: AP019585.1, strain: pvAJ020 Sequence ID: AP019584.1with a maximum score of 425 out of total scores 425, 99.56% identity with 97% query coverage. Moreover, there were no similarities with other related organisms, such as HTLV-I and II (human T-cell lymphotropic viruses), PTLV (primary T-cell lymphotropic viruses), and HERV-K K (human endogenous retrovirus).

### Phylogenetic analysis based on gag Gene

**Figure 6:**
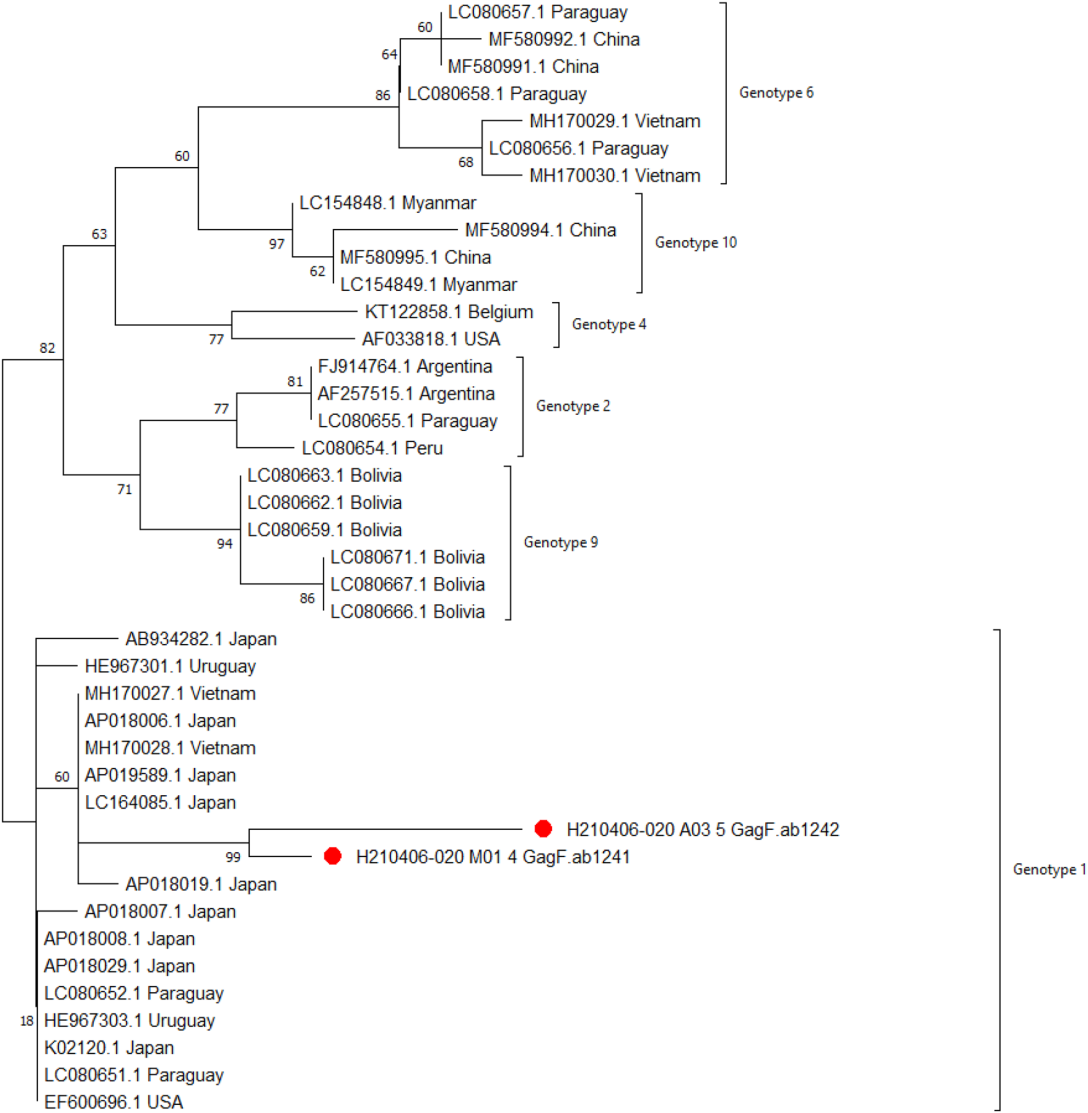
The phylogenetic tree of gag 4 and gag 5 is indicated by red dots. Next to the branches is the proportion of trees in which the related taxa clustered together. The initial tree(s) for the heuristic search were automatically generated by applying the Neighbor-Join and BioNJ algorithms to a matrix of pairwise distances computed using the Tamura-Nei model and then picking the topology with the highest log-likelihood value. The branch lengths are measured in the number of substitutions per site, and the tree is depicted to scale. Each descending clade’s proportion of sites with at least one unambiguous base in at least one sequence is shown next to each internal node in the tree.

The phylogenetic analysis involved 41 nucleotide sequences. There was a total of 245 positions in the final dataset. Evolutionary analyses were conducted in MEGA X^42^. The evolutionary history was inferred by using the Maximum Likelihood method and Tamura-Nei model^43^. The tree with the highest log likelihood (− 714.63) is shown in Fig. 6. The phylogenetic trees showed corresponding topologies, supported by high bootstrap values. The analysis of the tree showed six sequence clusters, designated as 1,2, 4, 6, 9, and 10 Genotypes. The gag 4 and gag 5 samples of the current study were assigned to genotype 1.

## Discussion

There are several well-recognized risk factors for breast cancer, but the main causes are still unidentified. According to recent findings, some viruses are involved in breast cancer and have oncogenic potential, such as MMTV (mouse mammary tumor virus), HPVs (human papilloma viruses), EBV (Epstein–Barr virus), and bovine leukemia virus (BLV)^44^. The relationship between breast cancer and BLV has inadequate evidence. However, recent findings from different countries showed varying percentages of BLV prevalence in breast cancer,*i.e.,*74% in Berkeley California^9^, 40.5%in Colombia^6^, 44% in Birmingham, Alabama, Pennsylvania, Ohio, Oakland, California^11^, 59% in California^10^, 80% in Australian women^45^, 34% in women in Texas^46^, 38% in California^4^, and 30.5% in South Brazil^47^, and the highest percentage of BLV prevalence was found at 95.9% in Minas Gerais, Brazil^48^. The association of BLV with breast cancer is still controversial, but the recent studies discussed above strongly suggest the significance of the relationship between breast cancer and BLV. Although even stronger findings are required to remark on this declaration, *i.e*., the presence of BLV, its association with breast cancer with larger sample size, and its route of transmission. If any relationship existed, it would have a momentous consequence on the global communal, financial, political sector and reveal the risk of BLV in Pakistan.

In the current study, we found that BLV was associated with breast cancer, with a 26.8% BLV prevalence. A 10% prevalence has been found in samples without cancer. These current findings indicated a correlation between the presence of the BLV genes and breast cancer (*odds ratio* = 0.3889; confidence interval = 1,18; p = 0.0029). To obtain reliable results, special precautions were taken to avoid cross-contamination of DNA, and nested-PCR was used for accurate, sensitive, and reliable *tax* and *gag* gene amplification. The resultant sequencing results with a high similarity index with the GenBank sequences show the accuracy of the results of the current study.

All the studies from different countries have variable percentages of BLV and breast cancer relationship, possibly due to the different lifestyle, diet, nutrition, exercise, environment, exposure to radiation, breastfeeding, age, genetic background, history, number of the sample size used, techniques applied for detection of the prevalence of BLV. Whatever the percentage of BLV detected by any study reveals the need to consider BLV as one of the risk factors for breast cancer. Consequently, along with the other risk factors for breast cancer, BLV requires special consent from all scientists globally. Animal diseases are more common than ever infecting people, resulting in a minimum of 2.5 billion diseases and 2.7 million annual fatalities.

Worldwide, breast cancer has an eminent morbidity and mortality rate, as it is a neoplastic disease among females. The query of the prospective danger of BLV for humans is an old but exceedingly topical focus of scientific debate. The possible risk of BLV to humans and the association of milk utilization with breast cancer have been studied earlier^49^. It is thought that approximately twelve percent of human cancers are due to viruses^50^. BLV surface glycoprotein (gp51) of the viral envelope was identified by immunohistochemistry in human breast cancer^51^ according to studies in Colombia. Californian scientists first noticed the regulatory gene (tax) of BLV in human breast cancer in 2014^11^. In American women's breast tissue, BLV DNA was discovered^11^ and was associated with breast cancer^10^. Moreover, one of the recent studies in Panama showed fifty-nine percent of the cancerous samples and thirty-eight percent of the precancerous samples by identifying the gag gene^12^.

The results from the current study confirm the presence of the BLV genome in the breast tissues of women in Pakistan and show a statistically significant positive association between the virus and breast cancer in this population. This association could be due to the high raw milk consumption in Pakistan. In comparison to treated milk, the requirement for untreated milk is high. Consequently, unprocessed milk and its product are commonly consumed in Pakistan, *i.e.,* almost more than 97% in Pakistan. The first report on the presence of BIV and BLV was based on a cattle population of Pakistan. Seroassays indicated p26 anti-BIV positivity in zero livestock cattle, and three *Bubalus buballis* were found to be positive for anti-BLV antibodies^52^. In addition, OIE in 2009 reported the absence of BLV from Pakistan^53^. Another study in 2019 reported that the prevalence of BLV in northwestern Pakistan was 20%^54^ and recently in 2021 the seropositivity of BLV was reported as 24.25% from Punjab (Pakistan)^55^. However, there is a need to reveal the route of BLV transmission in humans. Milk and meat consumption from BLV-infected animals could be a possible transmission route. Thus, there is a need for further exploration of BLV association with humans and its transmission route.

## Conclusion

The motivation behind the present study was to determine the relationship between breast cancer and the bovine leukemia virus within Pakistan, and the association of BLV with breast cancer in Pakistani women was found to be 26.8%. This significant association is not negligible. Thus, there is a need to control BLV, and further investigation about its risk related to public health is highly required.

## Supplementary Information

Supplementary Information.
