# Supplementary material for: Molecular investigation of possible relationships concerning bovine leukemia virus and breast cancer
Source: Sci Rep. 2022 Mar 9;12:4161. doi: 10.1038/s41598-022-08181-5 (PMC8907172; doi:10.1038/s41598-022-08181-5)
Supplement: Supplementary file 1 — Supplementary Information. [file 41598_2022_8181_MOESM1_ESM.docx]

Supplementary File

**Supplementary Table 1 (ST.1): PCR Reaction Mixture for *Tax* and *gag* Gene 30 μl**

| First run | | Second Run | |
| --- | --- | --- | --- |
| Reagents | **Conc.** | **Reagents** | **Conc.** |
| Template DNA | 2 ng | Product of First Run | 5μl |
| Reverse Outer Primer | 50pmol | Reverse Inner Primer | 50pmol |
| Forward Outer Primer | 50pmol | Forward Inner Primer | 50pmol |
| Master Mix | 2x | Master Mix | 2x |
| Nuclease Free Water | 10μl | Nuclease Free Water | 10.5μl |

**PCR Conditions Optimization**

Optimized Conditions of gag outer gene PCR

| S.No. | Steps | Temperature (°C) | Time Duration | Cycle |
| --- | --- | --- | --- | --- |
| 1 | **Initial denaturation** | 95 | 2 min | 1x |
| 2 | **Denaturation** | 94 | 30 sec |  |
| 3 | **Annealing** | 54 | 60 sec | 30x |
| 4 | **Extension** | 72 | 45 sec |  |
| 5 | **Final extension** | 72 | 10 min | 1x |

Optimized Conditions of gag inner gene PCR

| S.No. | Steps | Temperature (°C) | Time Duration | Cycle |
| --- | --- | --- | --- | --- |
| 1 | **Initial denaturation** | 94 | 2 min | 1x |
| 2 | **Denaturation** | 95 | 30 sec |  |
| 3 | **Annealing** | 56 | 60 sec | 30x |
| 4 | **Extension** | 72 | 45 sec |  |
| 5 | **Final extension** | 72 | 10 min | 1x |

Optimized Conditions of tax outer gene PCR

| S.No. | Steps | Temperature (°C) | Time Duration | Cycle |
| --- | --- | --- | --- | --- |
| 1 | **Initial denaturation** | 95 | 5 min | 1x |
| 2 | **Denaturation** | 94 | 45 sec |  |
| 3 | **Annealing** | 50 | 45 sec | 40x |
| 4 | **Extension** | 72 | 45 sec |  |
| 5 | **Final extension** | 72 | 10 min | 1x |

Optimized Conditions of gag inner gene PCR

| S.No. | Steps | Temperature (°C) | Time Duration | Cycle |
| --- | --- | --- | --- | --- |
| 1 | **Initial denaturation** | 95 | 5 min | 1x |
| 2 | **Denaturation** | 94 | 30 sec |  |
| 3 | **Annealing** | 55 | 60 sec | 40x |
| 4 | **Extension** | 72 | 45 sec |  |
| 5 | **Final extension** | 72 | 10 min | 1x |

**Supplementary Table 2 (ST.2):** The table of breast cancer distribution based on type

|  | Fibro | FD | GM | IMC | IDC | ILC | PT |
| --- | --- | --- | --- | --- | --- | --- | --- |
| BLV Positive | 155 | 11 | 0 | 0 | 559 | 2 | 2 |
| BLV Negative | 1372 | 133 | 83 | 45 | 313 | 20 | 15 |
| Total samples | 1527 | 114 | 83 | 45 | 872 | 22 | 17 |

*Fibro=Fibroadenoma, FD=Fibrocystic Disease, IMC=Invasive Mammary Carcinoma, IDC=Invasive Ductal Carcinoma, GM=Granulamatous Mastitis, ILC=Invasive Lobular Carcinoma, PT=Phyllodes Tumor

**Supplementary Table 3 (ST.3):** Fisher's exact test and chi-square test

| **P value, statistical significance** | | | |
| --- | --- | --- | --- |
| Test | Fisher's exact test |  |  |
|  |  |  |  |
| P value | 0.0029 |  |  |
| P value summary | ** |  |  |
| One- or two-sided | Two-sided |  |  |
| Statistically significant (P < 0.05)? | Yes |  |  |
|  |  |  |  |
| Effect size | Value | 95% CI |  |
|  |  |  |  |
| Relative Risk | 0.3972 | 0.2079 to 0.7559 |  |
| Reciprocal of relative risk | 2.518 | 1.323 to 4.811 |  |
|  |  |  |  |
| Attributable risk (P1 - P2) | 0.02056 | 0.009344 to 0.03465 |  |
| NNT (reciprocal of attrib. risk) | 48.63 | 28.86 to 107 |  |
|  |  |  |  |
| Odds ratio | 0.3889 | 0.1928 to 0.7405 |  |
| Reciprocal of odds ratio | 2.571 | 1.35 to 5.186 |  |
|  |  |  |  |
| **Methods used to compute CIs** | | | |
| Relative Risk | Koopman asymptotic score |  |  |
| Attributable risk (P1 - P2) | Newcombe/Wilson with CC |  |  |
| Odds ratio | Baptista-Pike |  |  |
|  |  |  |  |
| Percentage of grand total | healthy | BLV |  |
| POSITIVE | 0.36% | 26.09% |  |
| NEGATIVE | 2.51% | 71.04% |  |

| **Chi-square test** | | | |
| --- | --- | --- | --- |
| Test | Chi-square |  |  |
| Chi-square, df | 8.24, 1 |  |  |
| z | 2.871 |  |  |
| P value | 0.0041 |  |  |
| P value summary | ** |  |  |
| One- or two-sided | Two-sided |  |  |
| Statistically significant (P < 0.05)? | Yes |  |  |


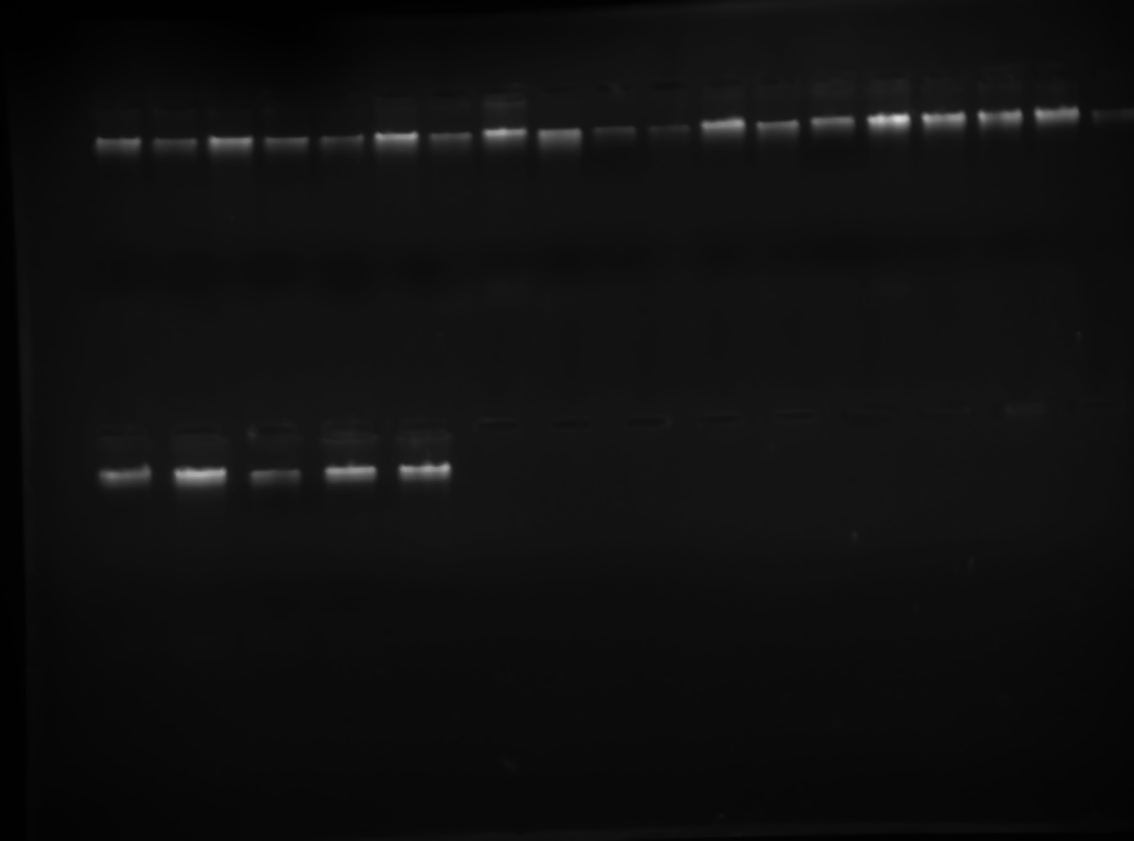


Figure SF. 1 Bands of the extracted whole genome DNA. It is an Un-cropped original Gel image.


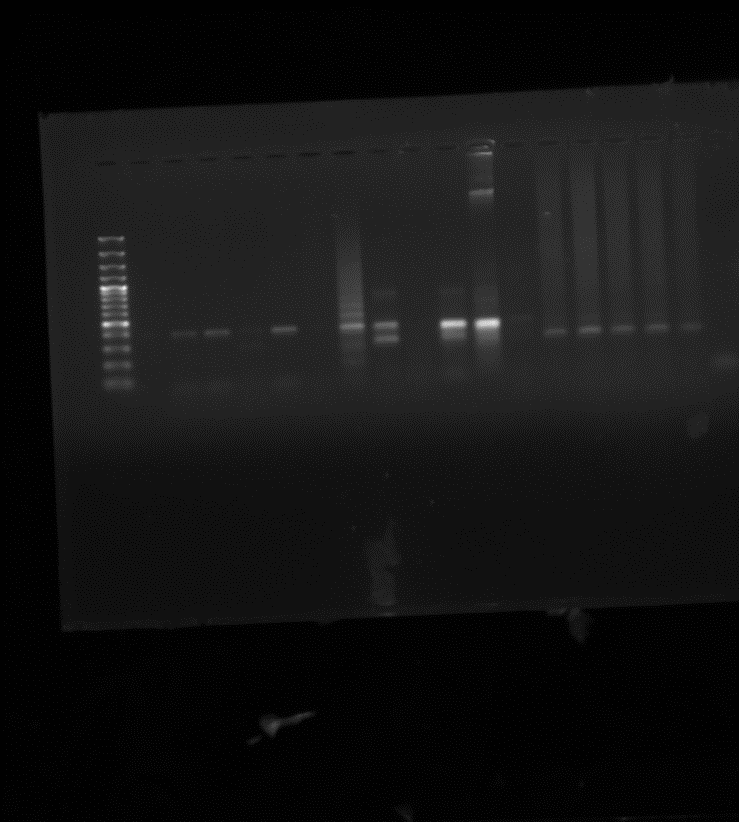


Figure SF. 2: This is the unlabbeled and raw figure presented as Fig 3 in main text. The figure shows the amplified gene fragments of the *gag* outer (385 bp) and *gag* inner genes (272) by nested PCR.
